# High-throughput transcriptome sequencing reveals the key stages of cardiovascular development in zebrafish embryos

**DOI:** 10.1186/s12864-022-08808-x

**Published:** 2022-08-13

**Authors:** Chune Zhou, Wei Zhao, Shuqiang Zhang, Junguo Ma, Yousef Sultan, Xiaoyu Li

**Affiliations:** 1 College of Life Science, Henan Normal University, Xinxiang, 453007 Henan China; 2 Department of Food Toxicology and Contaminants, National Research Centre, Dokki, Cairo, 12622 Egypt

**Keywords:** Transcriptome analysis, Cardiovascular system, Zebrafish embryos, Differentially expressed genes

## Abstract

**Background:**

The cardiovascular developmental process is a tightly regulated network involving multiple genes. The current understanding of the molecular mechanism behind cardiovascular development is insufficient and requires further research.

**Results:**

Transcriptome sequencing of three developmental stages in zebrafish embryos was performed and revealed three key cardiovascular developmental stages. Then, the differentially expressed genes (DEGs) involved in cardiovascular development were screened out. The three developmental stages were 18 (T1), 24 (T2), and 42 h post fertilization (hpf) (T3), and the three stages were confirmed by detecting differences in expression between cardiomyocyte and endothelial marker genes (*cmlc2, fli1)* using in situ hybridization, which represents the characteristics of cardiovascular development. Thousands of DEGs were identified using transcriptome analysis. Of them, 2605 DEGs were in T1-vs-T2, including 2003 up-regulated and 602 down-regulated genes, 6446 DEGs were in T1-vs-T3, consisting of 4608 up-regulated and 1838 down-regulated genes, and 3275 DEGs were in T2-vs-T3, including 2420 up-regulated and 855 down-regulated genes. There were 644 common DEGs and 167 common five-fold higher differentially expressed genes (HDEGs) identified, and Gene Ontology (GO) and Kyoto Encyclopedia of Genes and Genomes (KEGG) pathway enrichment analyses were performed using the Database for Annotation, Visualization and Integrated Discovery (DAVID). Significant differences was observed in the levels of gene expression among different developmental stages in multiple GO terms and KEGG pathways, such as cell migration to the midline involved in heart development, cardiovascular system development, circulatory system process for biological processes of GO terms; and cardiac muscle contraction, adrenergic signaling in cardiomyocytes for KEGG pathways. These results demonstrated that these three stages were important period for the development of the cardiovascular system. Lastly, we used quantitative real-time PCR (qPCR) to validate the reliability of RNA-sequencing by selecting 21 DEGs.

**Conclusions:**

These results demonstrated that these three stages represented the important periods for cardiovascular system development of zebrafish and some candidate genes was obtained and provided a solid foundation for additional functional studies of the DEGs.

**Supplementary Information:**

The online version contains supplementary material available at 10.1186/s12864-022-08808-x.

## Background

Proper vascularization is necessary for embryonic development and adult survival. In zebrafish, the cardiovascular system develops before other organ systems during embryogenesis, with circulation beginning approximately 24 h post-fertilization (hpf) [1, 2]. Cardiovascular development is a dynamic, spatiotemporally, and transcriptionally regulated process involving thousands of genes [3].

Zebrafish are increasingly popular in many fields of scientific research due to their rapid growth, relatively short reproductive period, and their ease of genetic manipulation [4]. They are a particularly good model animal for studying vascular development since they have transparent embryos, can be manipulated genetically [5], and their embryos can survive without blood circulation for approximately seven days post-fertilization. Therefore, it is easy to screen for vascular mutants.

In the present study, we focused on investigating important cardiovascular system-related genes during early embryogenesis, based on the zebrafish developmental model. The differentially-expressed genes (DEGs) among three key developmental stages (18, 24, and 42hpf) were analyzed using integrated transcriptome sequencing. The results demonstrated that these three stages were important for cardiovascular system development. This study provided a preliminary basis for additional exploration of DEG functions, particularly common DEGs.

## Results

### Characteristics of cardiovascular development at three developmental stages

**Fig. 1:**
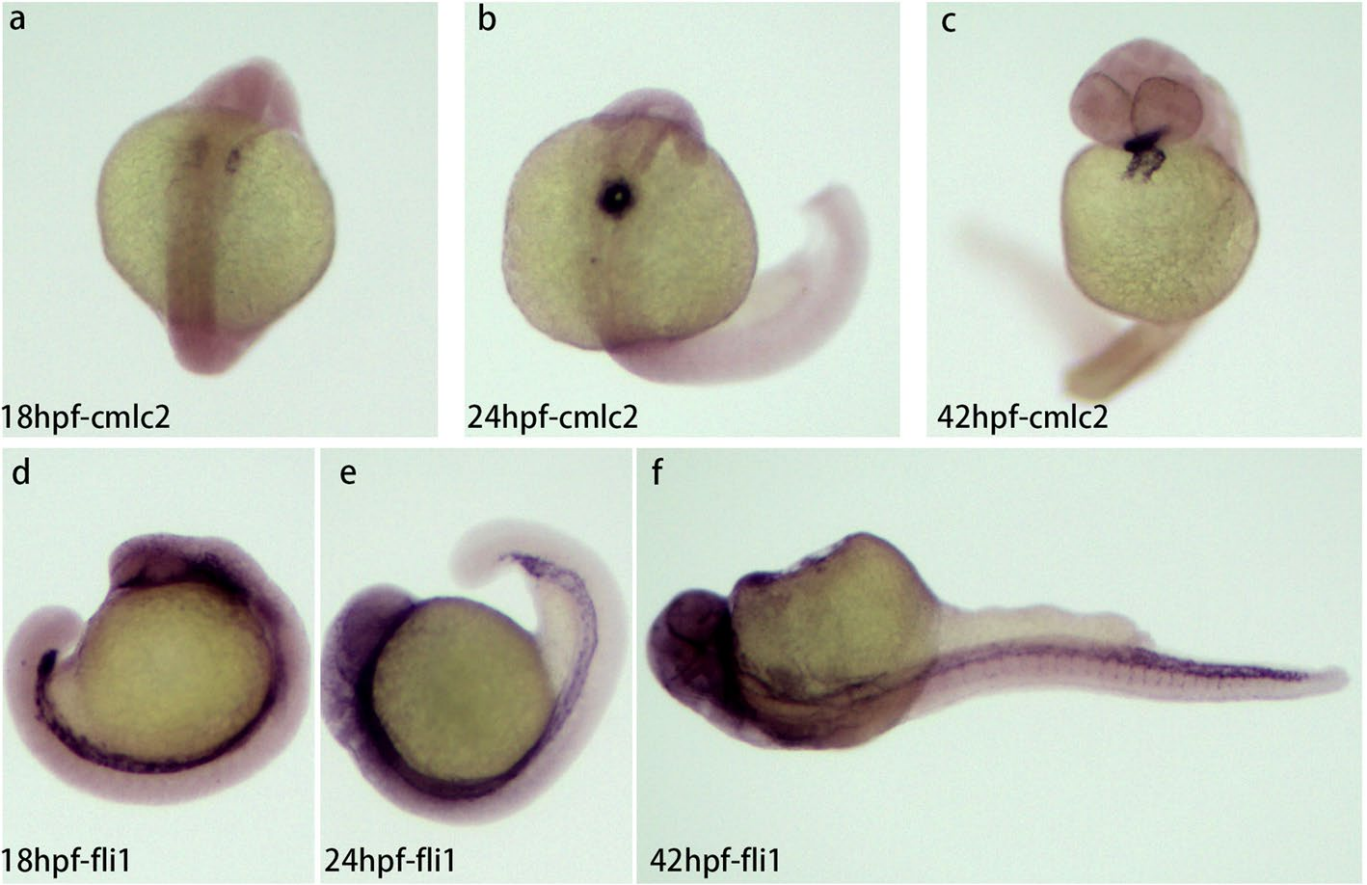
Expression of cardiomyocyte and endothelial marker genes (*cmlc2*, *fli1*) at three different stages of zebrafish embryo development (18, 24, 42 hpf). a-c Expression of *cmlc2* at 18hpf (**a**), 24hpf (**b**), and 42hpf (**c**). A pair of primordia were generated at 18 hpf, one on each side of the midline (**a**), The primordia on both sides fused to form a single linear heart tube at 24 hpf (**b**), the heart tube undergoes a loop and the heart chamber was visible (**c**). V, ventricle; A, atrial. d-f Expression of *fli1* at 18 hpf (**d**), 24 hpf (**e**). and 42hpf (**f**)

Three key developmental stages were selected, including 18, 24, and 42hpf (Fig. 1). The characteristics of cardiovascular development at three stages were demonstrated through in situ hybridization using the antisense probe of cardiomyocyte and endothelial marker genes (*cmlc2* and *fli1* respectively, the probe primers was listed in Supplementary Table 2). A pair of primordia were generated at 18 hpf, one on each side of the midline (Fig. 1a). The primordia on both sides fused to form a single linear heart tube at 24 hpf (Fig. 1b); this is when the heart begins to contract and blood starts to flow. By the time the embryo reaches 42 hpf, the heart tube undergoes looping morphogenesis, the heart chambers are visible, and the ventricle and atrium have been separated (Fig. 1c). The expression of endothelial marker genes (*fli1*) was only found in the main vessels at 18 hpf and 24 hpf embryos (Fig. 1d, e). However, when the embryo developed to 42 hpf, it was found in intersegmental vessels (ISVs), which connect the dorsal arteries and veins (Fig. 1f) and ensure that blood flows throughout the body. These results revealed that these three stages are so important for the development of the cardiovascular system that some genes of the cardiovascular system developmental network are likely active. Therefore, comparative transcriptome analysis across three stages can provide a foundation for further research into the molecular mechanisms of genes associated with specific biological processes.

### RNA sequencing and de novo transcriptome assembly

**Fig. 2:**
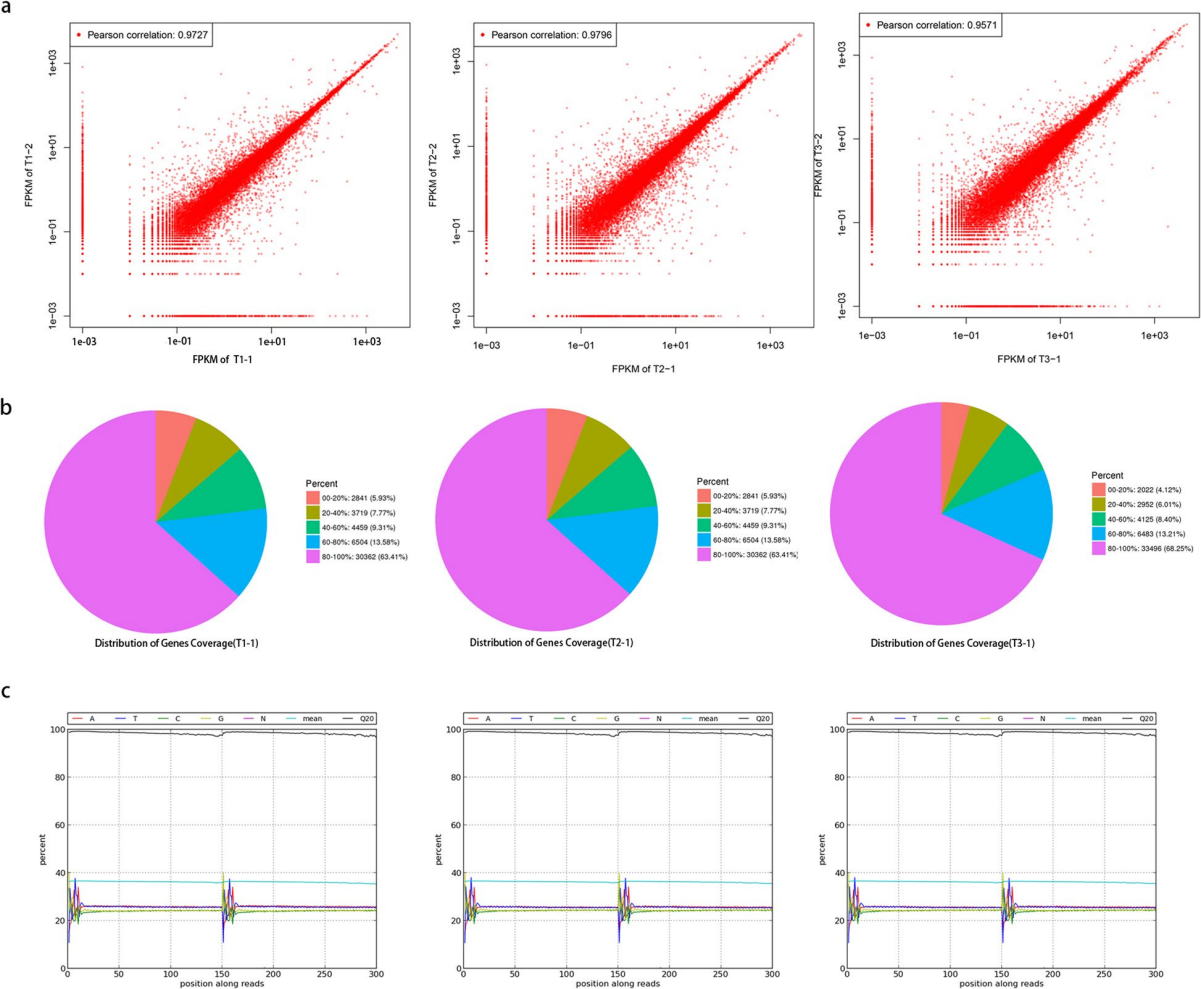
Quality analysis of transcriptome sequencing. **a** Correlation coefficient maps between different sequencing repeats. **b** Statistical maps of gene coverage. **c** Base composition and quality distributions

To identify gene transcription profiles of three key developmental stages, RNA sequencing was performed using an Illumina HiSeq2500 system. The percentage of bases of Q20 ≥ 20 reached 99% (Fig. 2c). The correlation coefficient between the two replicates exceeded 95% (Fig. 2a), demonstrating sufficient repeatability between the two parallel experiments. The 80%-100% gene coverage accounted for approximately 70% of total genes (Fig. 2b). These results demonstrate that the sequencing data was credible enough for further analysis.

### Identification of DEGs among three key developmental stages

**Fig. 3:**
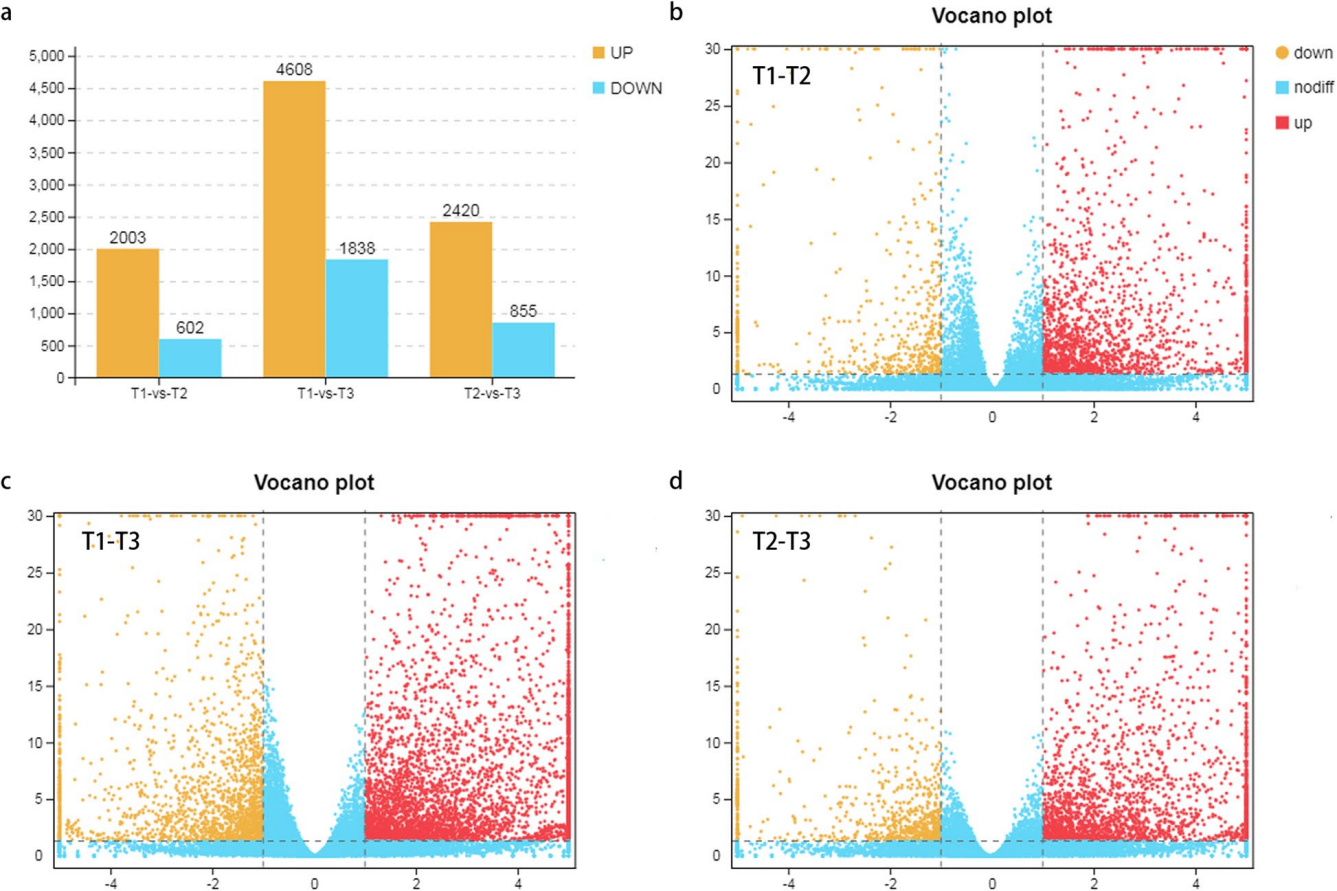
Analysis of DEGs in three stages of zebrafish embryo development. **a** Comparison of the number of up- and down-regulated genes. **b**-**d** Volcano plots between different groups. Yellow and red points represent up- and down-regulated genes, respectively, blue points represent genes with no differences

Thousands of DEGs were found to be up-regulated or down-regulated according to the result of transcriptome analysis across three different developmental stages. (Supplementary table 3, 4, 5). There were 2605 DEGs in T1-vs-T2, including 2003 up-regulated genes and 602 down-regulated genes, 3275 in T2-vs-T3, including 2420 up-regulated genes, and 855 down-regulated genes, and 6446 in T1-vs-T3, including 4608 up-regulated genes and 1838 down-regulated genes (Fig. 3a). T1-vs-T3 had the most DEGs compared to T1-vs-T2 and T2-vs-T3, while T1-vs-T2 had the fewest. Interestingly, there were more up-regulated DEGs than down-regulated DEGs in each comparative group. The number of DEGs up-regulated in T1-vs-T2 was 3.3 times higher than the number of down-regulated ones, 2.8 times higher in T2-vs-T3, and 2.5 times higher in T1-vs-T3. Volcano plots indicated that the number of up-regulated DEGs was significantly higher than the number of down-regulated DEGs (Fig. 3).

### GO term and KEGG pathway enrichment analysis of DEGs

**Fig. 4:**
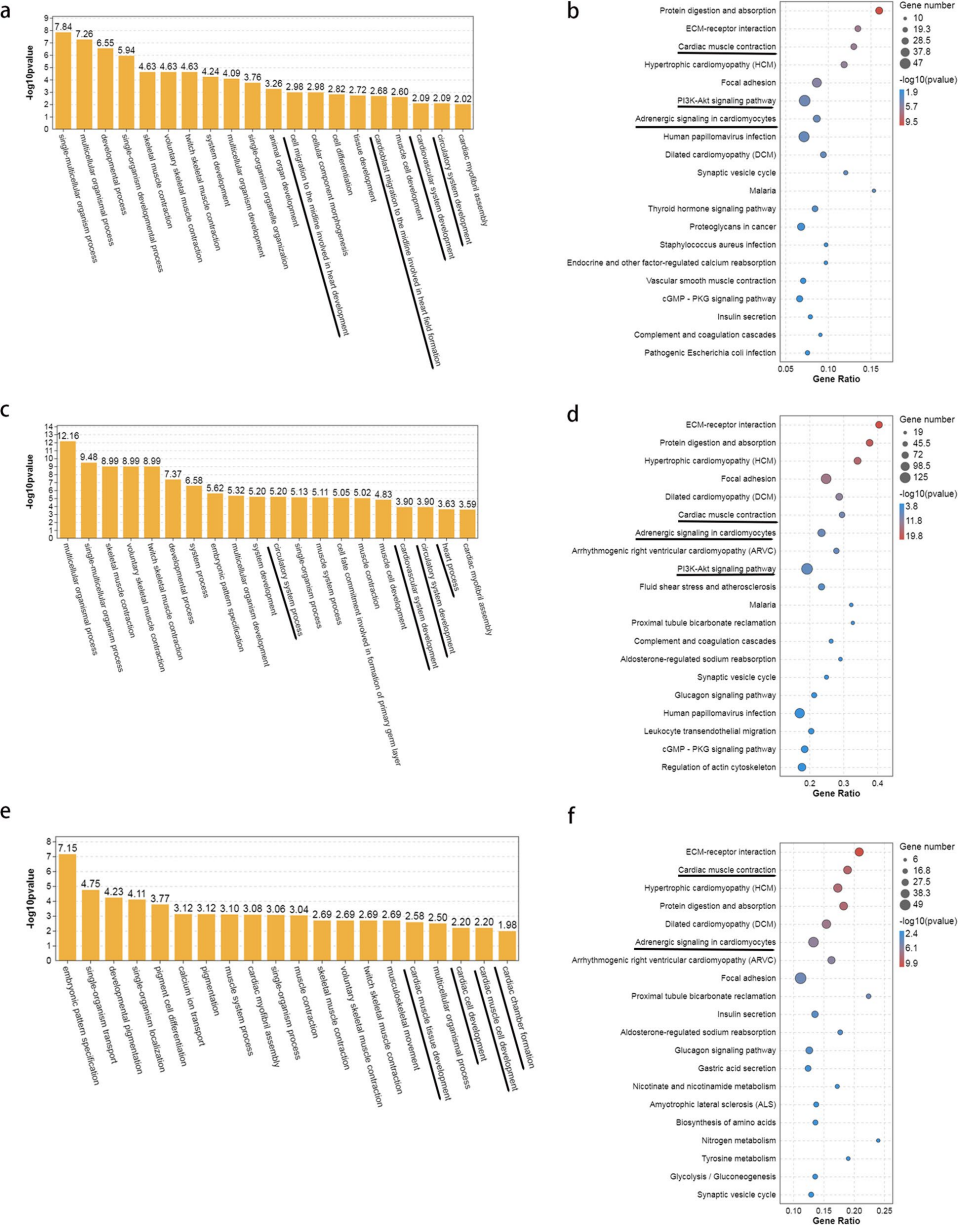
GO and KEGG enrichment analysis of DEGs between different stages of zebrafish embyro development. **a** GO enrichment analysis of DEGs between T1 and T2. **b** KEGG enrichment analysis of DEGs between T1 and T2. **c** GO enrichment analysis of DEGs between T1 and T3. **d** KEGG enrichment analysis of DEGs between T1 and T3. **e** GO enrichment analysis of DEGs between T2 and T3. **f** KEGG enrichment analysis of DEGs between T2 and T3

To further understand DEGs function during three different developmental stages, all DEGs were subjected to GO term analysis and KEGG pathway enrichment analysis [6] (Fig. 4). GO term analysis demonstrated that DEGs in T1-VS-T2 were primarily enriched in some biological processes (*p* < 0.05), two of them were cell migration to the midline involved in heart development (GO 0,003,318), cardioblast migration to the midline involved in heart field formation (GO 0,060,975) (Fig. 4a, Supplementary table 6). The results indicated that a pair of primordia on both sides migrated to the midline and fused to form an intermediate cone when embryos developed from 18 to 24hpf, which were consistent with the results of in situ hybridization of cardiomyocyte marker gene *cmlc2* (Fig. 1). Three of GO terms which enriched in T1-VS-T3 were circulatory system process (GO 0,003,013), cardiovascular system development (GO 0,072,358), and circulation system development (GO 0,072,359) (Fig. 4c, Supplementary table 7). This results indicated that the stages from 18 to 42hpf may be very important for the cardiovascular system development. Four terms enriched in biological processes in T2-VS-T3 were cardiac muscle tissue development (GO 0,051,179), cardiac cell development (GO 0,007,626), cardiac muscle cell development (GO 0,055,006) and cardiac chamber formation (GO 0,003,008) (Fig. 4e, Supplementary table 8). This results demonstrated that the cardiac muscle was developed and formed the chamber when embryos developed from 24 to 42hpf.

The results of KEGG analysis indicated that the DEGs across three groups were enriched in some pathways related to the circulatory system, such as cardiac muscle contraction, adrenergic signaling in cardiomyocytes and PI3K-AKT signaling pathway (Fig. 4 Supplementary table 9, 10, 11). The results demonstrated that the three stages were important for the cardiovascular system development, which provide the basis for screening the genes involved in cardiovascular system development.

### Identification of common DEGs in three stages

**Fig. 5:**
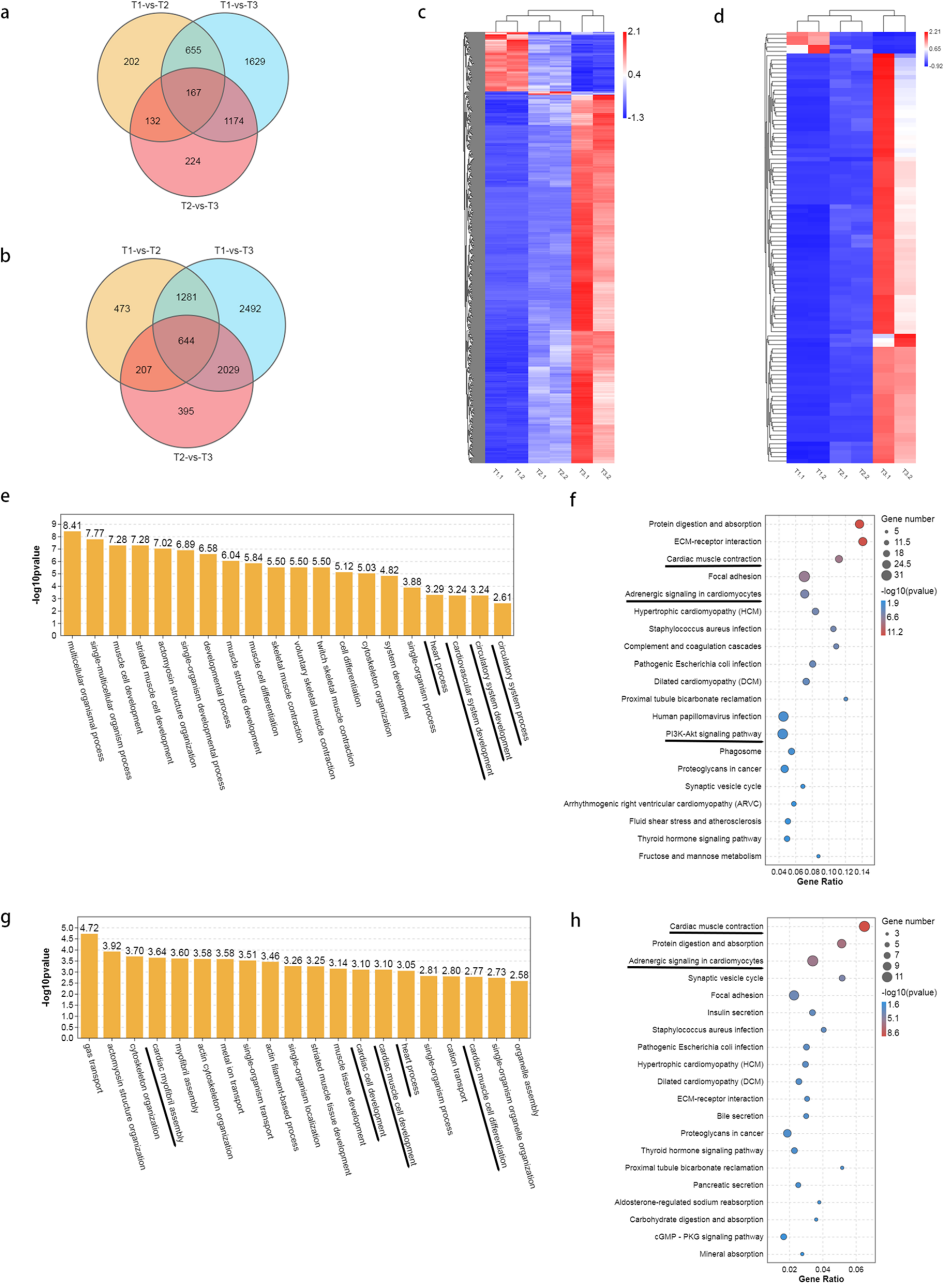
Common DEGs in three stages of zebrafish embryo development. **a** Common HDEGs were obtained in three stages. **b** Common DEGs were obtained in three stages. **c** Hierarchical clustering of 644 common DEGs. **d** Hierarchical clustering of 167 common HDEGs. **e** GO enrichment analysis of 644 common DEGs. **f** KEGG enrichment analysis of 644 common DEGs. **g** GO enrichment analysis of 167 common HDEGs. **h** KEGG enrichment analysis of 167 HDEGs

To further understand the DEG function in the three stages, common DEGs and common HDEGs of the three comparative groups were identified (Fig. 5a, b). There were 644 common DEGs (Fig. 5b); of them, 568 genes were up-regulated and 76 genes were down-regulated. There were 167 common HDEGs (Fig. 5a), including 161 up-regulated genes and 6 down-regulated genes. Interestingly, hierarchical clustering analysis of the DEGs based on normalized FPKM values indicated that common DEGs (Fig. 5c) and common HDEGs (Fig. 5d) were primarily up-regulated, while only a few were down-regulated. Additionally, differences in T1 vs T2 were less important than differences in T1 vs T3 and T2 vs T3.To further explore the role of DEGs in the three comparative groups, we performed KEGG pathway enrichment analysis on 644 common DEGs and 167 common HDEGs. The results of GO analysis demonstrated that 644 common DEGs were enriched in some terms related to heart development, such as heart process, cardiovascular system development, circulatory system development, circulatory system process (Fig. 5e, Supplementary table 12), while 167 common HDEGs in cardiac myofibril assembly, cardiac cell development, cardiac muscle cell development, heart process and cardiac muscle cell differentiation (Fig. 5g, Supplementary table 13). The results of KEGG enrichment analysis demonstrated both 644 common DEGs and 167 HDEGs were enriched the cardiovascular-related pathways, such as cardiac muscle contraction, adrenergic signaling in cardiomyocytes and PI3K-AKT signaling pathways (Fig. 5f, h, Supplementary table 14, 15). In conclusion, these three developmental stages were possibly crucial for cardiovascular development.

### Identification of cardiovascular-related DEGs

Expression profiles demonstrated that common DEGs were associated with multiple pathways. While common DEGs and HDEGs were most significantly enriched in the circulatory system, they were also involved in other pathways, such as protein digestion and absorption and adrenergic signal transduction in cardiomyocytes. Therefore, we selected the genes enriched in the circulatory system and cardiovascular disease for hierarchical cluster analysis (Fig. S1), and the results showed that there were 42 DEGs listed in Table 1 and 11 HDEGs listed in Table 2.

**Table 1:** The cardiovascular development and disease-related genes in common DEGs

No	Gene ID	Gene symbol
1	ENSDARG00000001870	atp1a1a
2	ENSDARG00000004931	slc8a3
3	ENSDARG00000008772	cacng1a
4	ENSDARG00000010472	atp1a2a
5	ENSDARG00000013755	acnt3a
6	ENSDARG00000014804	cacna2d1a
7	ENSDARG00000018259	atp1a3a
8	ENSDARG00000019367	tgfb3
9	ENSDARG00000020574	atp2a1
10	ENSDARG00000020610	tnnt2a
11	ENSDARG00000024141	cav3
12	ENSDARG00000029764	mef2ca
13	ENSDARG00000032242	tnnt2c
14	ENSDARG00000032976	cmlc1
15	ENSDARG00000039173	ctslb
16	ENSDARG00000039832	gsta2
17	ENSDARG00000041799	gja1
18	ENSDARG00000042245	myl13
19	ENSDARG00000042428	gstt1
20	ENSDARG00000042552	cacna1s
21	ENSDARG00000053201	zgc:172,323
22	ENSDARG00000054931	ppp2r5b
23	ENSDARG00000058656	desma
24	ENSDARG00000062592	myl10
25	ENSDARG00000070923	zgc:158,296
26	ENSDARG00000075549	cdh5
27	ENSDARG00000075924	rapgef4
28	ENSDARG00000076833	atp1b1b
29	ENSDARG00000079564	myh7
30	ENSDARG00000087574	nox1
31	ENSDARG00000089806	si:dkey-239j18.3
32	ENSDARG00000103747	cav1
33	ENSDARG00000103969	smhc2
34	ENSDARG00000114031	myh7
35	ENSDARG00000114818	CU633479.5
36	ENSDARG00000115657	prkca
37	ENSDARG00000116713	si:ch73-265d7.2
38	MSTRG.11363	itgb1
39	MSTRG.1910	mef2c
40	MSTRG.25366	ppp2r3b
41	MSTRG.7667	acta1
42	MSTRG.8268	ctsv

**Table 2:** The cardiovascular development and disease-related genes in common HDEGs

No	Gene ID	Gene symbol
1	ENSDARG00000008772	cacng1
2	ENSDARG00000010472	atp1a2a
3	ENSDARG00000018259	atp1a3a
4	ENSDARG00000020574	atp2a1
5	ENSDARG00000032242	tnnt2c
6	ENSDARG00000032976	cmlc1
7	ENSDARG00000042245	myl13
8	ENSDARG00000042552	cacna1s
9	ENSDARG00000076833	atp1b1b
10	ENSDARG00000079564	myh7
11	MSTRG.7667	acta1

### qPCR validation

**Fig. 6:**
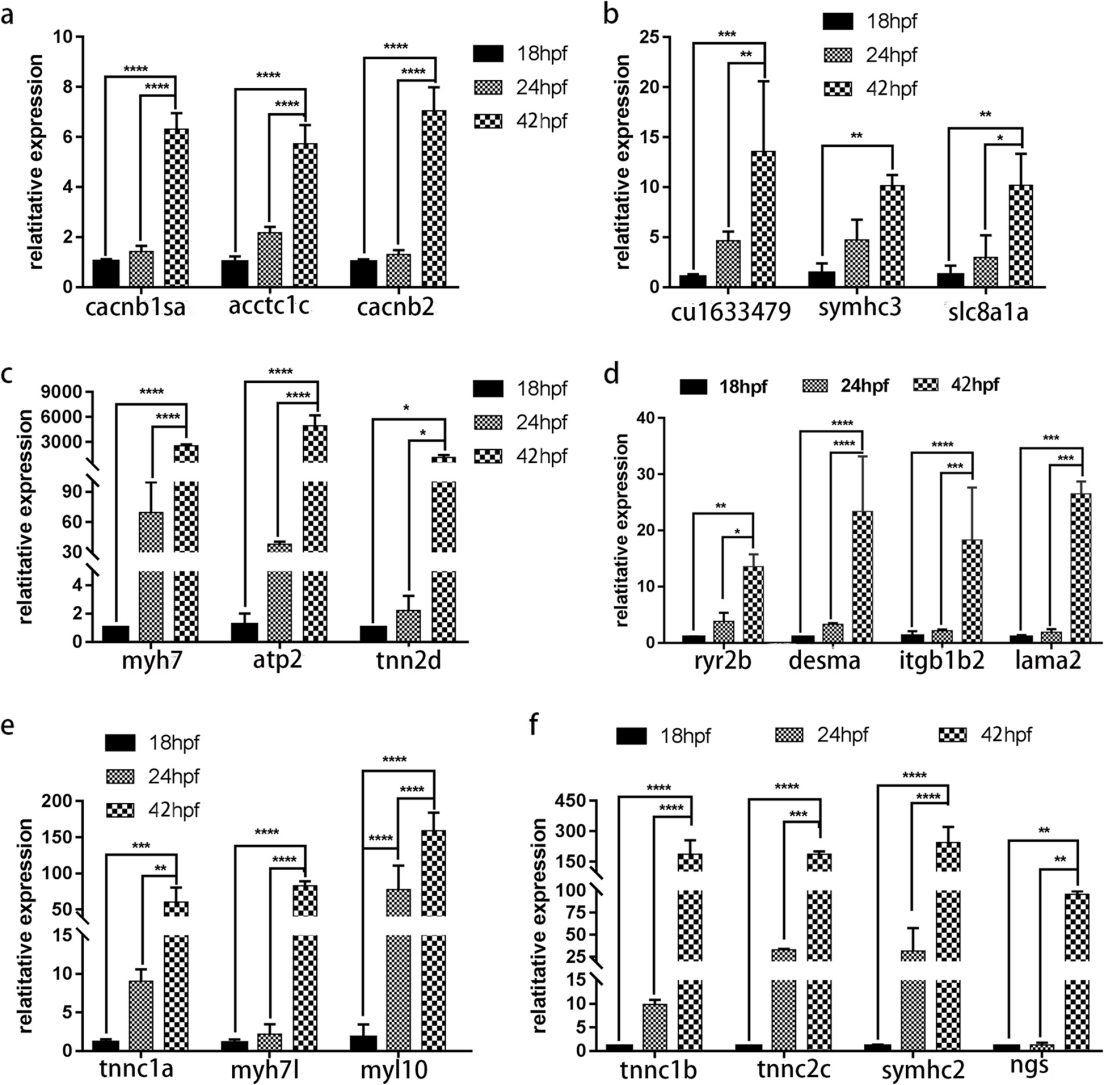
Validation of DEGs by qPCR. The expression of 18 hpf was set as 1, and the relative expression level of the other two stages (24 hpf and 42 hpf) was calculated for several genes. The asterisk indicates significantly different expression levels (*P < 0.05; **,*** and ****P < 0.01)

To validate the reliability of the RNA sequencing data, we selected 21 DEGs (*cacnb1sa*, *acctc1c*, *cacnb2*, *cu1633479*, symhc3, *slc8a1a*, *myh7*, *atp2*, *tnn2d*, *ryr2b*, *desma*, *itgb1b2*, *lama2*, *tnnc1b*, *tnn2c*, *tnnc1a*, *myh7l*, *myl10, tnnc2c, symhc2, ngs*), which were mainly related to cardiovascular development and cardiovascular disease pathways. The qPCR results demonstrated that the gene expression levels of these DEGs were similar to the RNA sequencing results, which confirmed the reliability of transcriptome analysis across three key cardiovascular stages (Fig. 6).

### Characterization of DEGs expression in different tissues

**Fig. 7:**
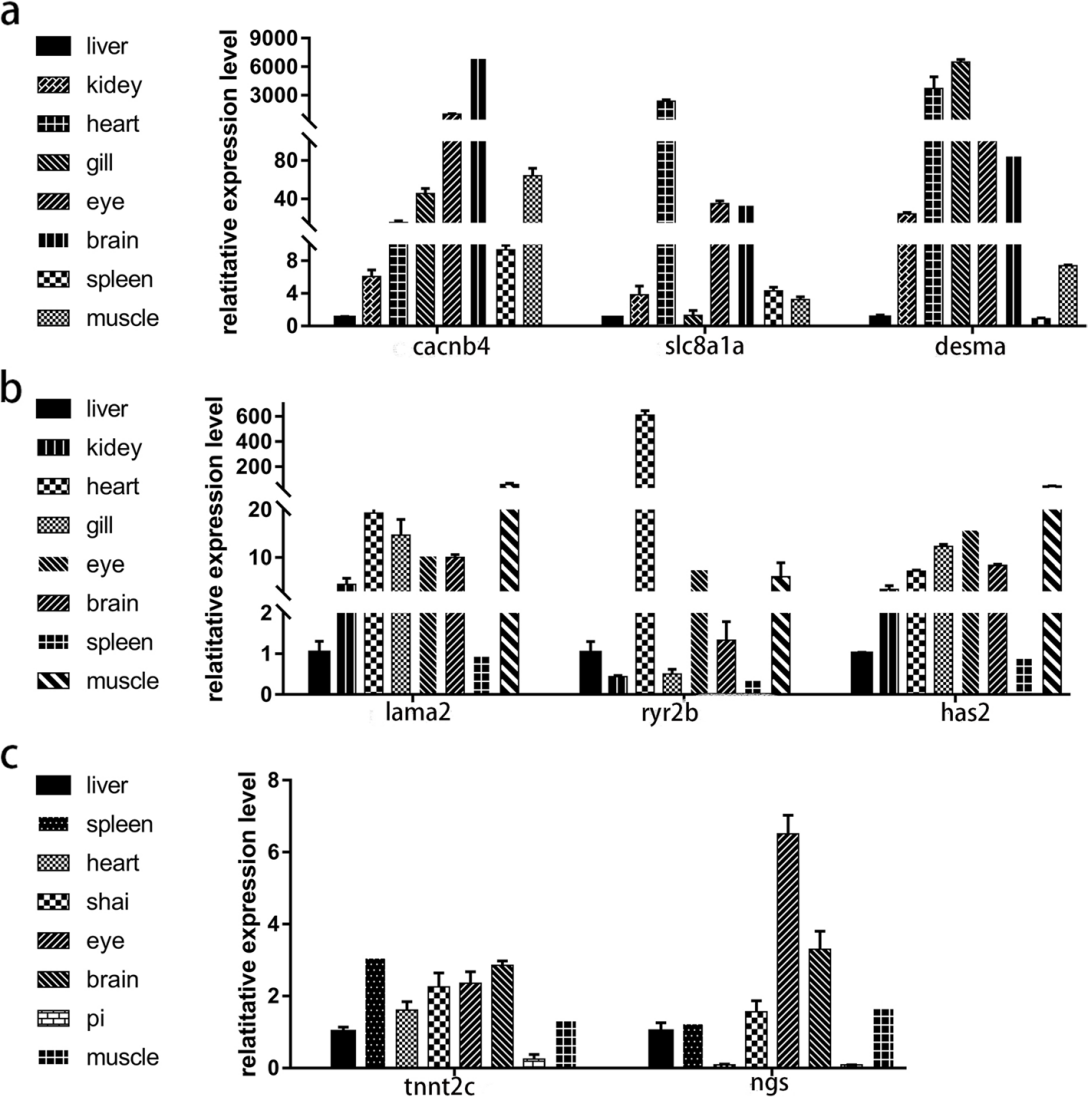
Expression of DEGs in different adult tissues

To further investigate the potential function of certain genes during cardiovascular development, their expression in different tissues was characterized. Most of them were highly expressed in the heart or muscle. For example, the expression level of *cacnb4* was highest in the brain, followed by the eyes; the expression of *Slc8a1a* was highest in the heart, followed by the eyes; the expression of *desma* was highest in the gills, followed by the heart; the expression of *lama2* was highest in the muscles, followed by the heart; the expression of *ryr2b* was highest in the heart, followed by the muscles and eyes; and the expression of *has2* was highest in the muscles, followed by the eyes and the heart (Fig. 7). The results suggest that these genes likely play a key role in cardiovascular development.

### Analysis of the spatial expression of DEGs

According to the results of qPCR verification in three stages, most genes showed an increasing trend across the three stages. The results of expression in different tissues found that *ryr2b*, *lama2*, *scl8a1a*, *has2*, and *desma* had higher levels of expression in the muscle or the heart than in other tissues. According to the annotation of the ZFIN database, *cntn2* was weakly expressed in the intersegmental vessels, so we chose these 6 genes to detect the spatial expression by the whole embryo in situ hybridization. The results demonstrated that *cntn2* expressed weakly in intersegmental vessels and strongly expressed in nerve tissues (Fig. S3). *Desma* was expressed in myotomes, hapaxial muscles, pectoral fin muscles, head muscles, and the heart (Fig. S2). *Has2* was mainly expressed in the heart, posterior somites, and tail bud (Fig. S4). *Lama2* was mainly expressed in the somites (Fig. S5), *Ryr2b* was mainly expressed in the heart (Fig. S6), and *Slc8a1a* was mainly expressed in the heart (Fig. S7).

## Discussion

### Transcriptome analysis in zebrafish embryogenesis

The rapid development of high throughput sequencing technology has allowed us to better understand the mechanism of various biological processes, including those of zebrafish. Previous transcriptome analysis primarily focused on early developmental stages [4, 7–9]. Results demonstrated that stage-specific genes are typically preferentially expressed during these stages and that these genes could be used as molecular markers to distinguish one stage from another [9]. This indicates that stage-preferential genes could have close relationships with biological processes. Since the morphological changes of the heart and blood vessels are significant at 18 hpf, 24 hpf, and 42 hpf in zebrafish embryos, the genes preferentially expressed during these three stages must be related to the development of heart and blood vessels. The aim of this study was to screen out genes related to cardiovascular development through transcriptome sequencing of these three stages.

### Important genes involved in cardiovascular development were identified

Transcriptome analysis of three developmental stages allowed us to screen out 42 genes that could be involved in cardiovascular development. Of these genes, *atp1a2a* regulates zebrafish right-left patterning [10] and plays a key role in establishing proper cardiac laterality in zebrafish [11]. Additionally, certain genes screened through the transcriptome analysis are involved in the cardiovascular developmental process, including cardiac contractility [11–14], cardiomyocyte differentiation [14], vascular stability [15], hematopoiesis [16], PAAs (The pharyngeal arch arteries) development [17] and heart development [18]. Some genes are also expressed in the heart [17, 19], but their functions are largely unknown and require additional study.

In this study, we used whole-mount in situ hybridization to identify the expression of 6 genes in zebrafish embryos. Of these, *desmin* was expressed in the head muscles, trunk muscles, pectoral fin muscles, in developing hearts and fins. These results are consistent with previous reports [20, 21]. *Desmin* has a muscle-specific expression pattern, and is expressed in smooth muscles, skeletal muscles, and myocardium [20]. However, its function in developing hearts is still unclear and requires further research. We found that *cntn2* was mainly primarily expressed in neurons and weakly expressed in intersegmental blood vessels. These results are consistent with those of previous studies [21]. Other research has reported that *cntn2* is involved in branchiomotor neuron migration [22–24], spinal cord regeneration [25], and axon fasciculation [26]. Nevertheless, the role of *cntn2* in intersegmental blood vessels has yet not been reported. Since it is expressed in intersegmental blood vessels, it could play a role in the development of intersegmental blood vessels, but this requires additional study. We determined that *lama2* was primarily expressed in the trunk muscles, which was consistent with previous studies [27, 28]. *Laminins* are the main components of basement members and are required for the development of embryonic and adult tissues [29]. It consists of three chains (alpha, beta, and gamma) which are encoded by different genes. They have different tissue-specific expression patterns and have different functions, which are described in previous reports [28]. Existing reports have demonstrated that *laminins* plays several roles in adults and embryos, including blood vessel formation [30], notochord formation [31], neuron migration [32], and neurogenesis [33, 34]. Interestingly, *laminin* alpha chains have overlapping roles in the formation of notochords and blood vessels [30]. However, little is known about the role of *lama2* in blood vessel formation. Therefore, this requires further study. In addition, our results also showed that *ryr2b* was dominantly expressed in the heart, which is consistent with previous studies [35]. However, its function in the heart has not yet been reported, and further research is necessary. Additionally, results of the heart-specific expression patterns of two genes detected in this study (*slc8a1a* and *has2*) was consistent with previous reports in which *slc8a1a* was reported to be required for proper cardiac morphogenesis, while *Has2* plays a key role in vasculature formation [36].

In conclusion, we screened out the DEGs from three key developmental stages of zebrafish embryonic cardiovascular system using transcriptome analysis, some genes, including *slc8a1a* and *has2*, were previously reported to be involved in cardiovascular development. Some genes were expressed in cardiovascular development, but their function is unknown and need more study in the future. To this end, our research provides more candidate genes for future work and to better understand the role of these genes in embryonic cardiovascular development.

## Methods

### Animal husbandry and embryo collection

Zebrafish (Danio rerio) were purchased from the Institute of Hydrobiology, Chinese Academy of Science (Wuhan, China). Wild-type (AB line) zebrafish were bred and maintained according to standard procedures [37] and staged as described [38]. All animal processing was approved by the Institutional Animal Care and Use Committee of Henan Normal University.

### RNA extraction, library construction, and sequencing

Total RNA was isolated using a Trizol reagent kit (Cwbiotech, Beijing, China) according to the manufacturer’s instructions. The resulting RNA was further sequenced using Illumina HiSeq2500 by Gene Denovo Biotechnology Co. (Guangzhou, China).

### Data filtering and de novo assembly

Raw reads containing adapters or low-quality bases produced from the sequencing machines were first filtered using fastp [39]. Reads mapped to the ribosome RNA (rRNA) database using the short reads alignment tool Bowtie2 [40] (version 2.2.8) were removed. The remaining reads were mapped to the reference genome using HISAT2. 2.4 [41]. The mapped reads from each sample were assembled using StringTie v1.3.1 [42].

### DEGs analysis and function annotation

DEGs analysis was performed by DESeq2 [43] software across the three stages. Genes with false discovery rate (FDR) ≤ 0.05 and absolute fold change ≥ 2 were considered as DEGs. To analyze DEGs function among the 18, 24, and 42 hpf stages, all DEGs were mapped to terms in the Kyoto Encyclopedia of Genes and Genomes (KEGG) database (http://www.genome.jp/kegg/pathway.html) and the Gene Ontology database (http://www.geneontology.org/). GO terms and KEGG pathways with FDR < 0.05 were deemed as significantly enriched GO terms and KEGG terms.

### Quantitative real-time PCR (qRT-PCR) validation

To validate the reliability of the transcriptome sequencing, qPCR was performed for 21 DEGs. The primers were designed using Primer 5.0 and sequences were provided in Supplementary Table 1. Total RNA was extracted from 50 embryos or tissuse from two adult zebrafish. First strand cDNA was reversely transcripted from 1 μg RNA with HIFIScript 1st strand cDNA synthesis kit (Cwbiotich, China). The independent experiment was performed in triplicate. The *rpl13a* gene was used as the internal control. The reaction system (10 μl) comprised 0.2 μl cDNA,5 μl SYBR green master mix, 0.4 μl each primer (20 μM), and 4 μl nuclease-free water. The PCR conditions were as follows: 10 min at 95 °C, followed by 45 cycles at 95 °C for 15 s, 60 °C for 60 s, and a cooling stage at 4 °C. The 2^−ΔΔCt^ method was used to analyze expression levels.

### Expression profile of DEGs in different tissues

To characterize the expression profile of DEGs in different tissues, qPCR was performed and used eight tissues from adult zebrafish as templates. The reaction system and PCR conditions were the same as those above.

### Spatial expression of DEGs

To further verify their spatial expression, six genes likely involved in cardiovascular development were selected to determine their expression sites in embryo development via whole-mount in situ hybridization. The primers of the probe were presented in Supplementary Table 2. The PCR fragments were cloned into a pGEM-T easy vector and sequenced to verify whether the fragment was ligated to the vector. The antisense and sense probe were transcripted by SP6 or T7 to generate the DIG-labeled probe using the linearized vector, including PCR target fragments as a template. Whole-mount in situ hybridization was performed as previously described [44].

### Statistical analysis

SPSS 20.0 was used for statistical analysis. Data was presented as mean ± SEM. Difference between groups we analyzed using the Student’s t-test. *P* values < 0.05 were considered to be statistically significant.

## Supplementary Information

**Additional file 1: Fig. S1.** The genes involving in cardiovascular development screened from the common DEGs and HDEGs. **Fig. S2.** Spatial expression of desma by whole mount embryo in situ hybridization. **Fig. S3.** Spatial expression of cntn2 by whole mount embryo in situ hybridization. **Fig. S4.** Spatial expression of has2 by whole mount embryo in situ hybridization. **Fig. S5.** Spatial expression of lama2 by whole mount embryo in situ hybridization. **Fig. S6.** Spatial expression of ryr2b by whole mount embryo in situ hybridization. **Fig. S7.** Spatial expression of slc8a1a by whole mount embryo in situ hybridization.

**Additional file 2: Table S1.** The primers of validated genes.

**Additional file 3: Table S2.** The probe primers of genes.

**Additional file 4: Table S3.** The DEGs of T1 VS T2.

**Additional file 5: Table S4.** The DEGs of T1 VS T3.

**Additional file 6: Table S5.** The DEGs of T2 VS T3.

**Additional file 7: Table S6.** The enriched GO terms in DEGs of T1 vs T2.

**Additional file 8: Table S7.** The enriched GO terms in DEGs of T1 vs T3.

**Additional file 9: Table S8.** The enriched GO terms in DEGs of T2 vs T3.

**Additional file 10: Table S9.** The enriched KEGG pathways in DEGs of T1 vs T2.

**Additional file 11: Table S10.** The enriched KEGG pathways in DEGs of T1 vs T3.

**Additional file 12: Table S11.** The enriched KEGG pathways in DEGs of T2 vs T3.

**Additional file 13: Table S12.** The enriched GO terms in 644 common DEGs.

**Additional file 14: Table S13.** The enriched GO terms in 167 common HDEGs.

**Additional file 15: Table S14.** The enriched KEGG pathways in 644 common DEGs.

**Additional file 16: Table S15.** The enriched KEGG pathways in 167 common HDEGs.

## Data Availability

RNA-seq data sets in our study are available in the National Centerfor Biotechnology Information Gene Expression Omnibus under the accession number GSE207476.

## Abbreviations

DEGs: Differentially expressed genes
GO: Gene ontology
KEGG: Kyoto encyclopedia of genes
qPCR: Quantitative real-time polymerase chain reaction
