# Supplementary material for: High-throughput transcriptome sequencing reveals the key stages of cardiovascular development in zebrafish embryos
Source: BMC Genomics. 2022 Aug 13;23:587. doi: 10.1186/s12864-022-08808-x (PMC9375324; doi:10.1186/s12864-022-08808-x)
Supplement: Supplementary file 1 — Additional file 1: Fig. S1. The genes involving in cardiovascular development screened from the common DEGs and HDEGs. Fig. S2. Spatial expression of desma by whole mount embryo in situ hybridization. Fig. S3. Spatial expression of cntn2 by whole mount embryo in situ hybridization. Fig. S4. Spatial expression of has2 by whole mount embryo in situ hybridization. Fig. S5. Spatial expression of lama2 by whole mount embryo in situ hybridization. Fig. S6. Spatial expression of ryr2b by whole mount embryo in situ hybridization. Fig. S7. Spatial expression of slc8a1a by whole mount embryo in situ hybridization. [file 12864_2022_8808_MOESM1_ESM.zip › Supplementary figure.pdf]

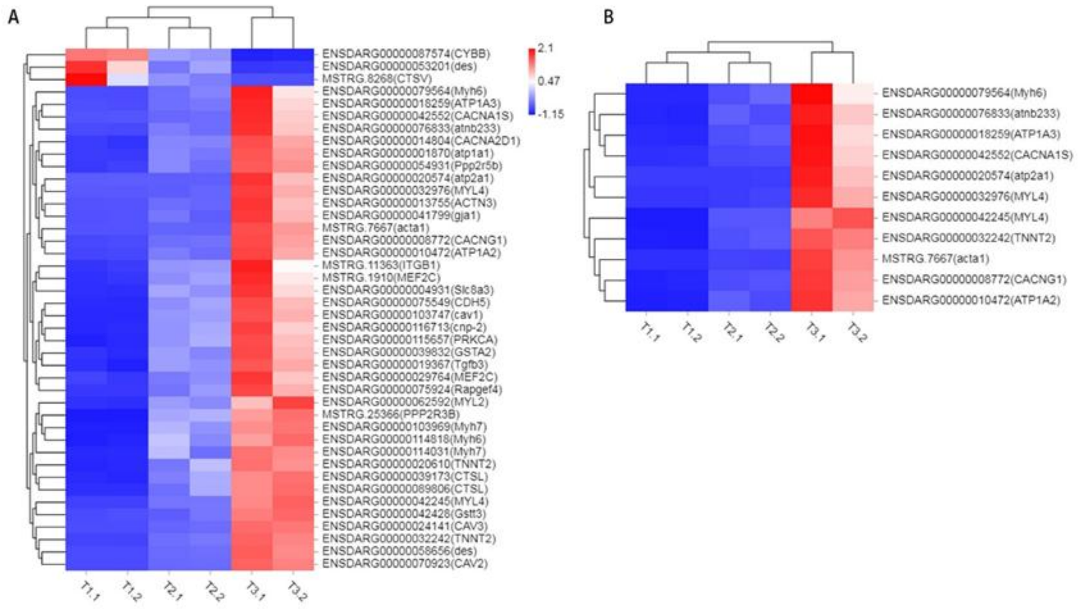

Fig. S1 The genes involving in cardiovascular development screened from the common DEGs and HDEGs. (A) Hierarchial clustering of 42 genes screened in common DEGs. (B) Hierarchial clustering of 11 genes screening from the HDEGs.

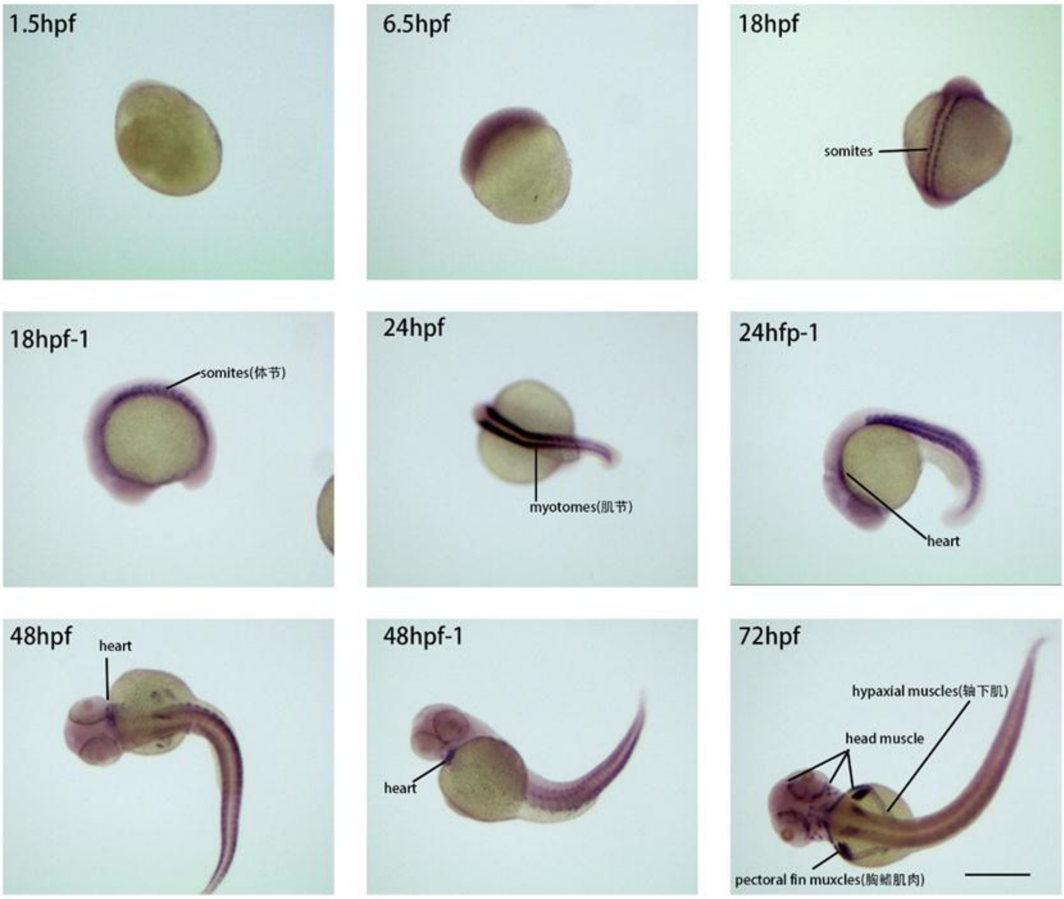

Fig. S2 Spatial expression of *desma* by whole mount embryo *in situ* hybridization

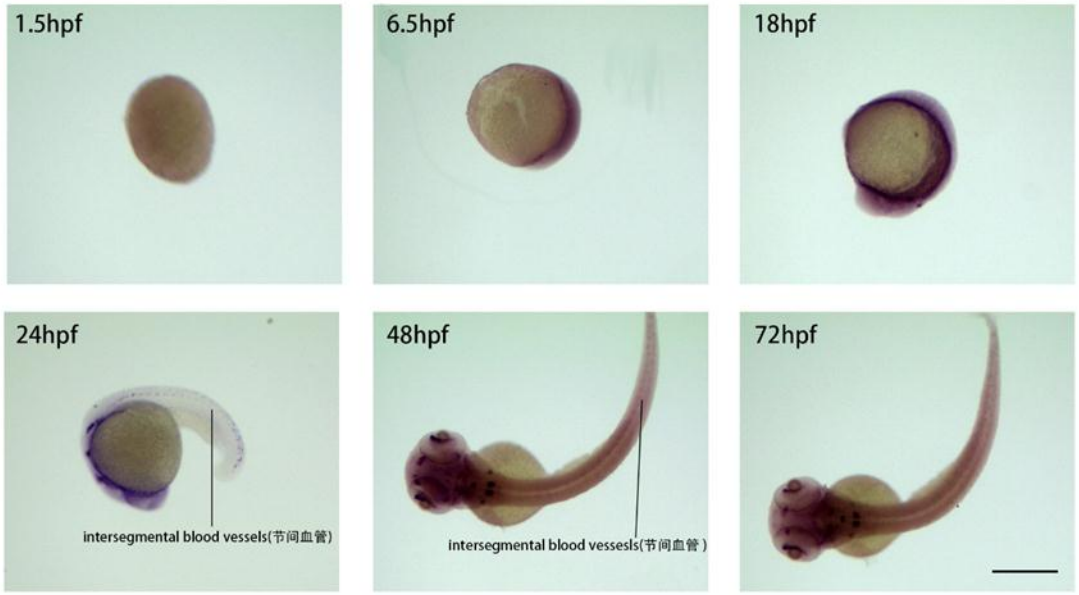

Fig. S3 Spatial expression of *cntn2* by whole mount embryo *in situ* hybridization

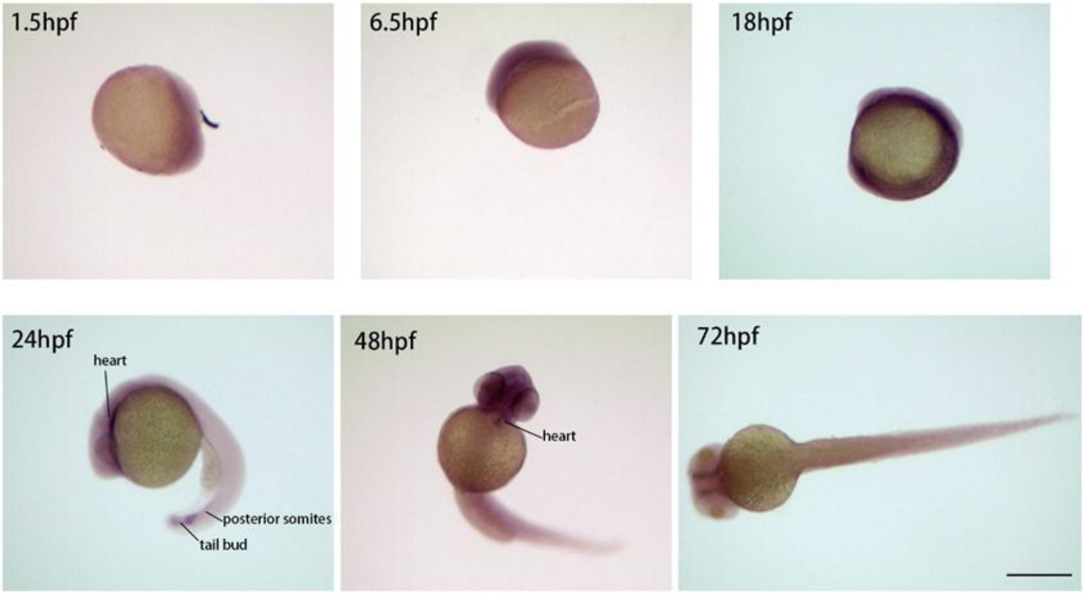

Fig. S4 Spatial expression of *has2* by whole mount embryo *in situ* hybridization

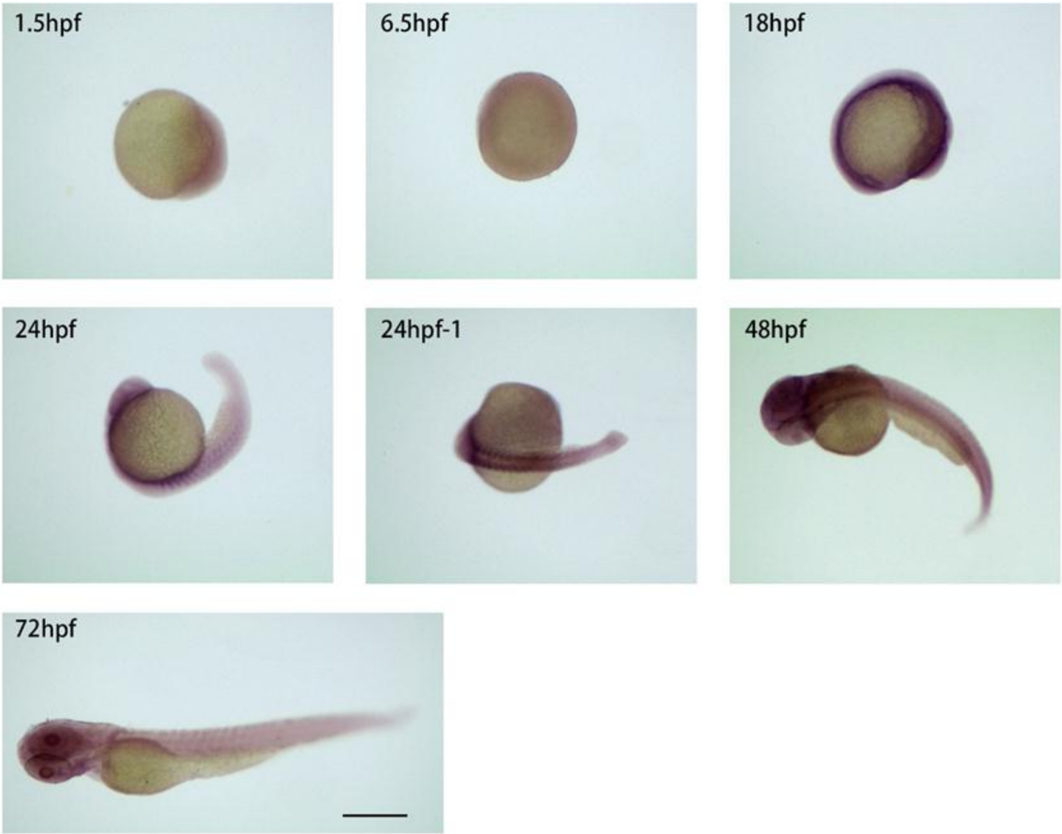

Fig. S5 Spatial expression of *lama2* by whole mount embryo *in situ* hybridization

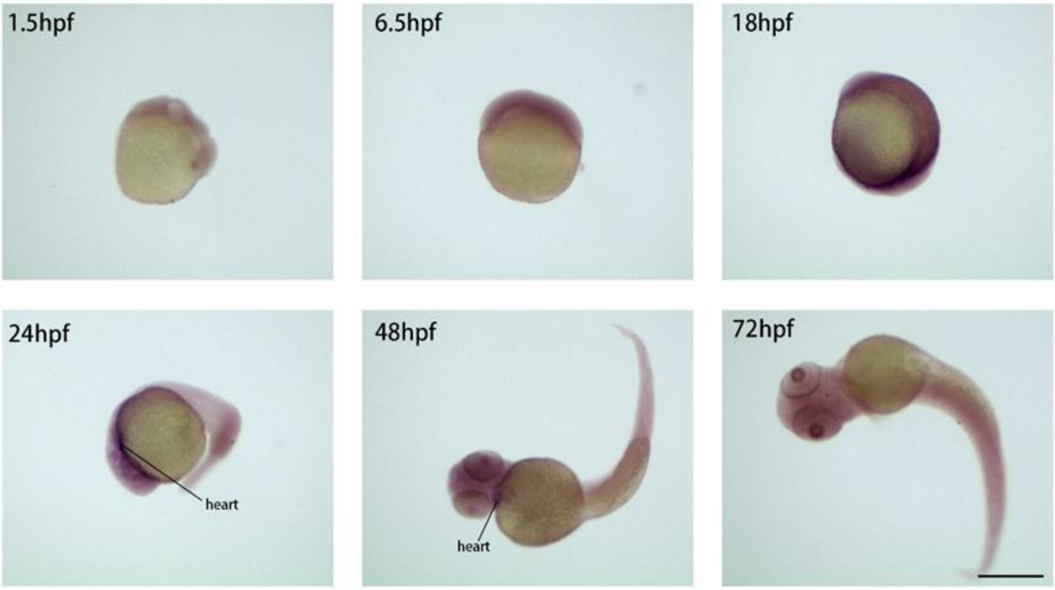

Fig. S6 Spatial expression of *ryr2b* by whole mount embryo *in situ* hybridization

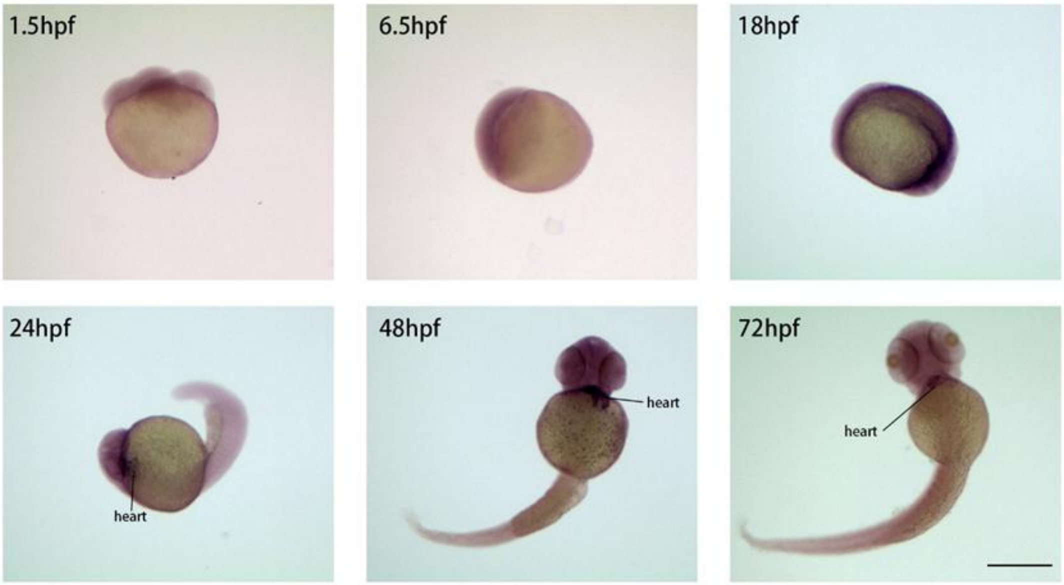

Fig. S7 Spatial expression of *slc8a1a* by whole mount embryo *in situ* hybridization
